# Revisiting Scrofula: An Entity Not to Forget in Migrants’ Health

**DOI:** 10.7759/cureus.40012

**Published:** 2023-06-05

**Authors:** Sara Neves, Fábio Videira Santos

**Affiliations:** 1 Department of Infectious Diseases, Centro Hospitalar Universitário de Santo António, Porto, PRT; 2 Department of Infectious Diseases, Instituto Português de Oncologia do Porto, Porto, PRT

**Keywords:** extrapulmonary tuberculosis, migrants, lymphadenitis, lymph node, tuberculosis

## Abstract

Tuberculous lymphadenitis (TBL) is one of the most common presentations of extrapulmonary tuberculosis (EPTB). The particularity of this presentation is the difficulty in establishing a definitive diagnosis, as clinical manifestations and imaging may be unspecific.

We describe a case of tuberculous cervical lymphadenitis in a young male from Pakistan, a high-burden tuberculosis country. We intend to raise awareness of this entity, given the high index of suspicion required to diagnose it, which can lead to delays in appropriate treatment, potentially increasing the morbidity and mortality of affected patients. Increased awareness is especially important in immigrants, in whom tuberculosis cases continue to increase, exposing the need for easy and equitable access to healthcare. A brief review of the subject is also presented.

## Introduction

In 2021, 6.4 million people were diagnosed and notified with tuberculosis (TB) globally. Of these, 17% were cases of extrapulmonary tuberculosis (EPTB) [1]. In Europe, of all TB cases reported in 2020, 21.5% were EPTB, with Portugal being one of eight countries with an EPTB proportion above 30% [2].

Tuberculous lymphadenitis (TBL) constitutes nearly 35% of all EPTB cases and most frequently affects cervical lymph nodes (60%-90%), a condition historically known as scrofula [3,4]. Scrofula is considered the local manifestation of a systemic disease that has disseminated to the lymph nodes, reflecting the reactivation of latent TB. However, reports have suggested that TB cervical lymphadenitis can occur through primary infection of the adenoids and tonsils, indicating that the pathogenesis of the disease remains unclear [5].

Most cases of TBL in high-income countries affect young immigrants from tuberculosis-endemic areas [3,6]. The disease has a female preponderance, and the peak age of onset is 30-40 years [7]. It usually presents as an isolated nontender painless lymphadenopathy with progressive enlargement, with systemic symptoms being uncommon [7]. The main complications seen when cervical lymph nodes are affected are fistulization, ulceration, and abscess formation.

The diagnosis of TBL is difficult, as it relies on a high index of suspicion from clinicians, who may not be familiar with its presentation. This lack of awareness can lead to delays in appropriate treatment, potentially increasing the morbidity and mortality of affected patients, some of whom might end up becoming an active source of transmission in the community through concomitant pulmonary involvement [3]. Once the cause is presumed, fine-needle aspiration (FNA) is a safer, less invasive procedure to collect biological samples compared with excisional biopsy [7]. For treatment in adults, the internationally accepted first-line regimen should be offered, which consists of two months of isoniazid, rifampicin, ethambutol, and pyrazinamide, followed by at least four months of isoniazid and rifampicin [8].

## Case presentation

A 24-year-old male from Pakistan, living in Porto, Portugal, for two years, working as a kitchen helper, with no past medical history or chronic medication, came to the emergency department due to a tumefaction on his right supraclavicular region. The patient first noticed the tumor two months before coming to the hospital and described a progressive enlargement, with an additional presentation of inflammatory signs such as pain, redness, and heat. He denied any systemic symptoms, specifically fever, night sweats, weight loss, shortness of breath, or coughing. He indicated having no contact with people who were sick and had no knowledge of cases of TB in his family.

**Figure 1:**
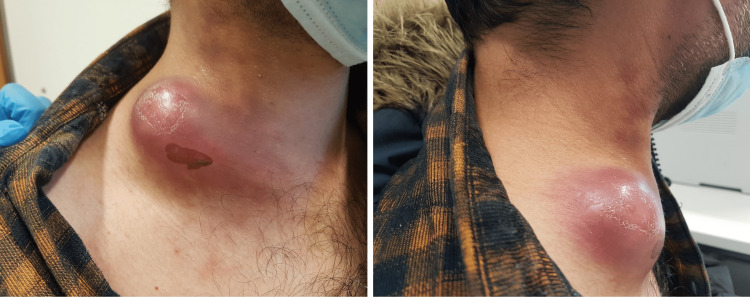
Enlarged right cervical lymph node

**Figure 2:**
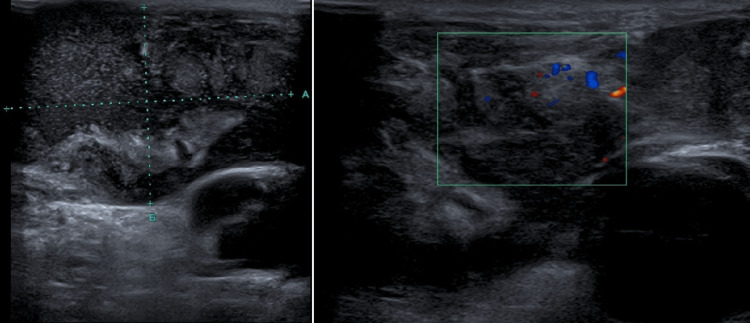
Ultrasound of the lesion showing a heterogenic structure with vascularization

On physical examination, the patient presented with a right cervical tumor measuring approximately 6 cm in diameter, which was warm and tender, with erythema, subtle fluctuation, and no evidence of exudates or fistulas (Figure 1). An ultrasound of the lesion revealed a heterogenic structure (5.6 × 3.6 cm) with areas of cystic appearance and others presenting vascularization, compatible with tuberculous lymphadenitis (Figure 2). FNA was performed, and samples were collected. HIV and viral hepatitis B and C serological tests were negative.

Given the suspicion of TB and the risk of pulmonary involvement, the patient was placed in an airborne infection isolation room, and sputum samples and a chest X-ray were requested. Unfortunately, the patient abandoned the emergency department before providing the samples and performing the X-ray, and public health authorities were promptly notified of the case.

The diagnosis was confirmed with both positive Ziehl-Neelsen (ZN) staining and polymerase chain reaction (PCR) of the *Mycobacterium tuberculosis* complex on the samples collected by FNA. One month later, culture was also positive, documenting susceptibility to first-line drugs.

Fortunately, the patient was later located and contacted by public health authorities, pulmonary involvement was excluded with a normal chest X-ray and negative ZN staining and PCR of the *Mycobacterium tuberculosis* complex on the sputum samples, and appropriate treatment was started.

## Discussion

In the presented case, the patient was a young immigrant from a country with a high burden of TB, which is consistent with the reported epidemiology of the disease in high-income countries [3,7]. He was living in Portugal for only two years, had no past medical history, and denied having contact with people who were sick, suggesting that the isolated TBL was probably due to the reactivation of latent TB. Contrary to the usually seen characteristics of TBL, the patient presented a tumor with inflammatory signs, and the ultrasound revealed a heterogenic structure with areas of cystic appearance evidencing abscess formation, one of the main complications seen in cervical TBL.

According to the World Health Organization (WHO), a diagnosis of TB is made based on a positive biological specimen by smear microscopy, culture, or a WHO-recommended rapid diagnostic test (e.g., Xpert MTB/RIF). Strong clinical evidence consistent with active TB in the absence of laboratory confirmation also qualifies as clinically diagnosed TB [8]. Biological samples may be obtained by FNA, which is a safe and inexpensive procedure with few complications [9]. In cases where the diagnosis is not achieved through FNA, excisional lymph node biopsy is the preferred alternative. Incision and drainage techniques have been associated with prolonged wound discharge and scarring and therefore are not recommended [10]. Once collected, specimens should be submitted to histology, acid-fast stain, nucleic acid amplification testing, and culture. Despite the growing use of WHO-approved molecular tests for the initial detection of the *Mycobacterium tuberculosis* complex, culture remains the gold-standard method to achieve a conclusive microbiological diagnosis and provides susceptibility testing [8]. In the presented case, there was a high risk of multidrug-resistant TB (MDR-TB) because Pakistan is a high-burden TB country and is estimated to have the third-highest prevalence of MDR-TB globally [1].

Whenever TB is suspected or diagnosed, it is essential to exclude pulmonary involvement to prevent disease transmission and other coinfections such as HIV, which can affect the patient’s prognosis. Treatment for EPTB does not differ from the well-known multidrug treatment regimens for the pulmonary forms of the disease, consisting of at least two months of isoniazid, rifampicin, ethambutol, and pyrazinamide, followed by four months of isoniazid and rifampicin [8]. Response to treatment is usually slow, and a paradoxical reaction may occur between three weeks and four months after treatment initiation, with an increase in lymph node size or enlargement of additional lymph nodes [7].

Considering the increasing globalization and the present European migrant crisis, the existence of a free, equitable, and accessible national health service has never been so vital to ensuring appropriate care for the most vulnerable populations. This case also emphasizes the importance of public health authorities in tracking patients with potentially transmissible diseases and handling their re-enrollment in required health services. Further studies concerning the cost-effectiveness of latent TB screening of immigrants from high-burden TB countries are needed, which could potentially prevent TB reactivation and disease transmission.

## Conclusions

It is important for physicians to note cases of TBL such as this one, given that symptoms and imaging can be undistinctive, despite being one of the most common presentations of EPTB. There was a high risk of multidrug-resistant TB in this case because Pakistan is a high-burden TB country and is reported to have the third-highest prevalence of MDR-TB globally. It is imperative that national health services are available and capable of ensuring easy access and equitable healthcare to the most vulnerable populations such as migrants and refugees to prevent public health crises.
